# Supplementary material for: Patients with Non-Alcoholic Fatty Liver Disease and Alcohol Dehydrogenase 1B/Aldehyde Dehydrogenase 2 Mutant Gene Have Higher Values of Serum Alanine Transaminase
Source: J Pers Med. 2023 Apr 28;13(5):758. doi: 10.3390/jpm13050758 (PMC10219542; doi:10.3390/jpm13050758)
Supplement: Supplementary file 1 [file jpm-13-00758-s001.zip › jpm-2352328-supplementary.pdf]

Supplement

Table S1 Correlation analysis between CAP, kPa and ADH1B allele

Coefficients[a]

Model		Unstandardized Coefficients		Standardized Coefficients		
		B	Std. Error	β	T	Sig.
CAP	（Constant）	133.818	52.888		2.530	0.014
	ADH1B_group	-2.086	19.699	-0.012	-0.106	0.916
	age	0.249	0.531	0.055	0.468	0.641
	gender	-17.168	13.088	-0.158	-1.312	0.195
	BMI	6.398	1.401	0.528	4.565	0.000
kPa	（Constant）	-9.115	5.468		-1.667	0.102
	ADH1B_group	-2.093	2.037	-0.128	-1.028	0.309
	age	0.111	0.055	0.257	2.016	0.049
	gender	0.492	1.353	0.048	0.364	0.718
	BMI	0.428	0.145	0.371	2.957	0.005

†adjusting factors of age, gender and BMI

Table S2 Correlation analysis between CAP, kPa and ALDH2 allele

Model		Unstandardized Coefficients		Standardized Coefficients		
		B	Std. Error	β	T	Sig.
CAP	（Constant）	137.167	54.127		2.534	0.014
	ALDH2_group	-5.696	13.161	-0.050	-0.433	0.667
	age	0.026	0.564	0.005	0.046	0.964
	gender	-12.928	14.159	-0.112	-0.913	0.365
	BMI	6.748	1.468	0.525	4.597	0.000
kPa	（Constant）	-10.131	5.116		-1.980	0.053
	ALDH2_group	-1.040	1.244	-0.105	-0.836	0.407
	age	0.108	0.053	0.254	2.027	0.047
	gender	0.771	1.338	0.076	0.576	0.567
	BMI	0.430	0.139	0.382	3.099	0.003

†adjusting factors of age, gender and BMI
